# Syntheses of Diheterocyclic Compounds Based on 2-Thioacetohydrazide-5,7-dimethyl-1,2,4-triazolo[1,5-a]-pyrimidine

**DOI:** 10.3390/molecules13061353

**Published:** 2008-06-13

**Authors:** Zu-Ming Liu, Qiong Chen, Chao-Nan Chen, Hai-Yang Tu, Guang-Fu Yang

**Affiliations:** Key Laboratory of Pesticide & Chemical Biology, Ministry of Education, College of Chemistry, Central China Normal University, Wuhan 430079, P. R. China; http://klpcb.ccnu.edu.cn/

**Keywords:** 1,2,4-Triazolo[1,5-a]pyrimidine, triazole, 1,3,4-oxadiazole, 1,3,4-thiadiazoles

## Abstract

The syntheses of some diheterocyclic compounds from 2-thioacetohydrazide-5,7-dimethyl-1,2,4-triazolo[1,5-a]pyrimidine (**1**) are described. Compound **1** can be converted into triazoles, 1,3,4-oxadiazoles, and 1,3,4-thiadiazoles. The structures of the intermediates and the target compounds were confirmed by ^1^H-NMR, MS and elemental analyses.

## Introduction

The study of nitrogen-containing heterocycles is currently a hot topic in pesticide chemistry [1,2,3,4,5,6,7]. In particular the chemistry of 1,2,4-triazolo[1,5-a]pyrimidine derivatives has been of considerable interest for many years [8]. In 1935, 5-methyl-7-hydroxy-1,2,4-triazolo[1,5-a]pyrimidine was found to be an excellent stabilizer for photographic emulsions. Since then, various derivatives of 1,2,4-triazolo[1,5-a]pyrimidine have found applications in pharmaceutical and agricultural chemistry and other areas [9,10]. On the other hand, a wide range of biological activities have been attributed to compounds containing 1,3,4-oxadiazole, 1,3,4-thiadiazole, 1,2,4-triazolo[3,4-b]-1,3,4-thiadiazole and 1,2,4-triazole rng systems [11,12,13,14]. If these heterocycles are introduced into the 1,2,4-triazolo[1,5-a]-pyrimidine ring, the linked diheterocyclic compounds might display interesting biological activity, so as a part of our research work aimed at searching for novel agrochemicals, our interest in diheterocyclic compounds containing 1,2,4-triazolo[1,5-a]pyrimidine moieties lead us to study the syntheses of the some diheterocyclic compounds based on the use of 2-thioacetohydrazide-5,7-dimethyl-1,2,4-triazolo[1,5-a]pyrimidine (**1**) as the starting material.

## Results and Discussion

2-Thioacetohydrazide-5,7-dimethyl-1,2,4-triazolo[1,5-a]pyrimidine (**1**) was prepared according to our previous procedure [5]. At room temperature, compound **1** reacted with CS_2_ in ethanol in the presence of potassium hydroxide, followed by treatment with hydrazine hydrate at reflux to afford compound **2**. The structure of **2** was confirmed by its ^1^H-NMR spectrum and elemental analysis. The NMR spectrum showed the methylene, amino and mercapto group protons as three singlets, at δ 4.56 ppm (SCH_2_), 5.66 ppm (NH_2_) and 13.67 ppm (SH), respectively.

Alkylation of **2** with alkyl halides afforded compounds **3** in good yields, whose structures were confirmed by their ^1^H-NMR spectra and elemental analysis. In ethanol solution and in the presence of HCl, **2** reacted with aromatic aldehydes to give a difused heterocyclic compound **4**. The pH value of the reaction solution influenced the yield of the product and experimental results showed that the best pH value was 4~6. In the ^1^H-NMR spectrum of compound **4**, the signals of the NH and methenyl (SCH) groups were observed as two singlets at δ 13.7~14.0 ppm (NH) and 9.50~ 10.90 ppm (SCH), respectively. The presence of these NH and SCH signals demonstrated the formation of 5,6-dihydrogen-1,2,4-triazolo[3,4-b]-1,3,4-thiadiazole moiety.

Compound **1** was also refluxed with carbon disulfide in ethanol in the presence of potassium hydroxide with subsequent treatment with hydrochloric acid. The ^1^H-NMR spectrum and elemental analysis data of the isolated product were consistent with the 1,3,4-oxadiazole structure **5**. Compound **5** reacted with alkyl halides in the presence of sodium hydroxide to afford compounds **6**. The reaction of **5** with alkyl halides is a typical nucleophilic substitution process and the experimental results indicated that the reactivity of the alkyl halide determined the reaction time and the yields. For example, intermediate **5** reacted with *p*-nitrobenzyl chloride under basic conditions at room temperature to give compound **6**a in yield 56% after 2 hours, but no product was observed when intermediate **5** reacted with 1-bromocyclohexane under the same conditions. Interestingly, compound **2** can also be obtained in 42% yield by refluxing compound **5** with hydrazine hydrate in methanol.

Heating compound **1** with benzoic acid in the presence of phosphoryl chloride afforded diheterocyclic compound **7** in 66% yield. The structure of **7** was established by ^1^H-NMR and elemental analysis. For example, the proton spectrum showed two methyl protons as two singlets at δ 2.64 ppm (5-CH_3_) and 2.73 (7-CH_3_), respectively. The SCH_2_ group protons and 6-protons in the 1,2,4-triazolo-[1,5-a]pyrimidine moiety displayed two singlets at δ 4.82 ppm (SCH_2_) and 6.76 ppm (6-H), respectively. The phenyl protons displayed a multiplet at δ 7.47-8.01 ppm (C_6_H_5_).

Furthermore, 2-thioacetohydrazide-5,7-dimethyl-1,2,4-triazolo[1,5-a]pyrimidine **1** can react with carbon disulfide in ethanol in presence of potassium hydroxide at room temperature with subsequent treatment with concentrated H_2_SO_4_ to afford a cyclization product **8** containing a 1,3,4-thiadiazole moiety, which was then alkylated with alkyl halides to give the corresponding compounds **9** in good yields. The factors affecting the yields and the reaction time for these alkylations of **8** were similar to those of the reaction of **5** with alkyl halides.

**Scheme 1:**
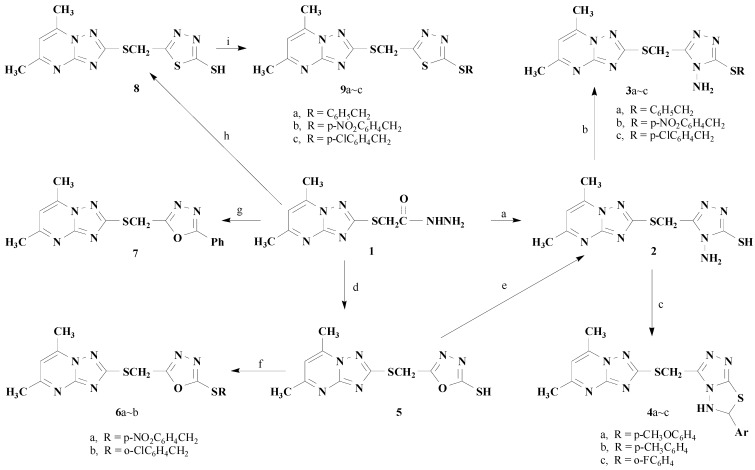
Synthesis of diheterocyclic compounds based on compound **1**.

a: (i) CS_2_, KOH, r.t; (ii) NH_2_NH_2_.H_2_O, reflux; b: RX, NaOH, r.t; c: ArCHO, HCl, reflux; d: CS_2_, KOH, reflux; e: NH_2_NH_2_.H_2_O, reflux; f: RX, NaOH, r.t; g: PhCOOH, POCl_3_, reflux; h: (i) CS_2_, KOH, r.t; (ii) H_2_SO_4_ (conc.); i: RX, NaOH, r.t.

## Experimental

### General

Melting points were measured with a Buchi melting point apparatus and are uncorrected. TLC was performed on Merck 60 F254 silica gel-coated aluminum sheets and spots were detected by UV light (254 nm).The ^1^H-NMR spectra were recorded on a Varian MERCURY-PLUS 400 instrument using the indicated solvents and TMS as an internal standard. MS spectra were recorded on a Hewlett-Packard 5988A instrument. Elemental analyses were performed on a Vario El III CHNS instrument. All solvents and materials were reagent grade and purified as required. 2-Thioacetohydrazide-5,7-dimethyl-1,2,4-triazolo[1,5-a]pyrimidine (**1**) was prepared as described in our published procedure [5].

### 4-Amino-3-(5,7-dimethyl-1,2,4-triazolo[1,5-a]pyrimidine-2-thiomethyl)-1,2,4-triazol-5-thiol (**2**)

*Method A*: To a solution of potassium hydroxide (5.0 g, 0.09 mol) and 2-thioacetohydrazide-5,7-dimethyl-1,2,4-triazolo[1,5-a]pyrimidine **(1**, 15.1 g, 0.06 mol) in ethanol (450 mL), carbon disulfide (10 mL, 0.16 mol) was added dropwise over a period of half hour at room temperature. The resulting mixture was stirred for 10 hours at room temperature and the precipitate formed was collected by filtration. After washing with ethanol and ethyl ether, the salt formed was dissolved in a solution of ethanol (300 mL) and hydrazine hydrate (85%, 15 mL, 0.26 mol), and then refluxed for 4 hours. After cooling, the mixture was filtered and the filtrate was poured into water (100 mL), acidified with HCl, and the precipitate thus formed was filtered off and crystallized from ethanol to give pure product **2** as a white solid in 38% yield, m.p. 228~229 ^o^C. ^1^H-NMR δ (DMSO-d_6_): 2.56 (s, 3H, CH_3_), 2.67 (s, 3H, CH_3_), 4.56 (s, 2H, SCH_2_), 5.66 (s, 2H, NH_2_), 7.12 (s, 1H, CH), 13.67 (s, 1H, SH); Anal. calcd. for C_10_H_12_N_8_S_2_ (308.37): C, 38.96; H, 3.90; N, 36.36. Found: C, 38.69; H, 3.77; N, 36.52.

*Method B*: Compound **5** (0.01 mol, 2.94 g), prepared as indicated below, was refluxed with an equimolar amount of 80% hydrazine hydrate in methanol (25 mL) for 2 hours. The solution was partially concentrated and cooled and the precipitate formed was filtered off and recrystallized from ethanol, yield: 27%.

### 5-Alkylthio-4-amino-3-(5,7-dimethyl-1,2,4-triazolo[1,5-a]pyrimidine-2-thiomethyl)-1,2,4–triazoles ***3a~c***

A mixture of RX (5.6 mmol) and methanol (or DMF, 5 mL) was added dropwise to a stirred solution of compound **2** (1.6 g, 5.0 mmol) and sodium hydroxide (0.2 g, 5.6 mmol) in water (15 mL). The resulting mixture was stirred at room temperature for 2 hours. The precipitate formed was filtered off and recrystallized from ethanol to give the pure title compounds in good yields.

**3a**: (R = C_6_H_5_CH_2_): m.p. 114~115 ^o^C; yield 82%; ^1^H-NMR δ(CDCl_3_): 2.60 (s, 3H, CH_3_), 2.70 (s, 3H, CH_3_), 4.29(s, 2H, SCH_2_), 4.55(s, 2H, SCH_2_), 6.71(s, 2H, NH_2_), 7.18(s, 1H, CH), 7.21 (m, 5H, C_6_H_5_); Anal. calcd. for C_17_H_18_N_8_S_2_ (398.50): C, 51.19; H, 4.52; N, 28.11; Found: C, 51.42; H, 4.37; N, 28.40.

**3b**: (R = *p*-NO_2_C_6_H_4_CH_2_): m.p. 227~228 ^o^C; yield 78%; ^1^H-NMR δ (CDCl_3_): 2.53 (s, 3H, CH_3_), 2.62 (s, 3H, CH_3_), 4.47 (s, 2H, SCH_2_), 4.56 (s, 2H, SCH_2_), 6.09 (s, 2H, NH_2_), 7.07 (s, 1H, CH), 7.59~8.08 (m, 4H, C_6_H_4_); Anal. calcd. for C_17_H_17_N_9_O_2_S_2_ (443.50): C, 45.99; H, 3.83; N, 28.41; Found: C, 45.67; H, 4.02; N, 28.76.

**3c**: (R = *p*-ClC_6_H_4_CH_2_): m.p. 175~176 ^o^C; yield 82.5%; ^1^H-NMR δ (CDCl_3_): 2.62 (s, 3H, CH_3_), 2.71 (s, 3H, CH_3_), 4.31 (s, 2H, SCH_2_), 4.56 (s, 2H, SCH_2_), 5.05 (s, 2H, NH_2_), 7.07 (s, 1H, CH), 7.19~7.27 (m, 4H, C_6_H_4_); Anal. calcd. for C_17_H_17_N_8_ClS_2_ (432.94): C, 47.12; H, 3.93; N, 25.87; Found: C, 46.88; H, 3.77; N, 26.24.

### 3-(5,7-Dimethyl-1,2,4-triazolo[1,5-a]pyrimidine-2-thiomethyl)-6-aryl-5,6-dihydrogen-1,2,4-triazolo-[3,4-b]-1,3,4-thiadiazoles ***4a-c***

The appropriate aryl aldehyde (3.3 mmol) was added to a solution of **2** (1.0 g, 3.3 mmol) and ethanol (60 mL) and the pH value of the mixture was then adjusted to 4~6 with an ethanol solution saturated with HCl gas. The resulting mixture was refluxed for 6~7 hours. After cooling, the precipitate was filtered off and crystallized from DMF/ethanol to give pure products as white solids in excellent yields.

**4a** (Ar = *p*-CH_3_OC_6_H_4_): m.p. 246~247 ^o^C; yield 80%; ^1^H-NMR δ (DMSO-d_6_): 2.51 (s, 3H, CH_3_), 2.55 (s, 3H, CH_3_), 3.81 (s, 3H, OCH_3_), 4.66 (s, 2H, SCH_2_), 7.01 (s, 1H, CH), 6.88~7.65 (m, 4H, C_6_H_4_), 9.56 (s, 1H, SCH), 13.84(s, 1H, NH); Anal. calcd. for C_18_H_18_N_8_OS_2_ (426.51): C, 50.64; H, 4.22; N, 26.26; Found: C, 50.49; H, 3.97; N, 26.50.

**4b** (Ar = *p*-CH_3_C_6_H_4_): m.p. 240~241 ^o^C; yield 86%; ^1^H-NMR δ (DMSO-d_6_): 2.50 (s, 3H, CH_3_), 2.52 (s, 3H, CH_3_), 2.54 (s, 3H, CH_3_), 4.66 (s, 2H, SCH_2_), 6.99 (s, 1H, CH), 7.12~7.56 (m, 4H, C_6_H_4_), 9.69 (s, 1H, SCH), 13.83 (s, 1H, NH); Anal. calcd. for C_18_H_18_N_8_S_2_ (410.51): C, 52.62; H, 4.38; N, 27.28; Found: C, 52.83; H, 4.59; N, 27.61.

**4c** (Ar = *o*-FC_6_H_4_): m.p. 237~239 ^o^C; yield 83.7%; ^1^H-NMR δ (DMSO-d_6_): 2.51 (s, 3H, CH_3_), 2.59 (s, 3H, CH_3_), 4.70 (s, 2H, SCH_2_), 7.02 (s, 1H, CH), 7.15~7.90 (m, 4H, C_6_H_4_), 10.43 (s, 1H, SCH), 13.91(s, 1H, NH); Anal. calcd. for C_17_H_15_N_8_FS_2_ (414.47): C, 49.22; H, 3.62; N, 27.02; Found: C, 49.50; H, 3.89; N, 27.33.

### 2-(5,7-Dimethyl-1,2,4-triazolo[1,5-a]pyrimidine-2-thiomethyl)-1,3,4-oxadiazol-5-thiol *(**5**)*

Compound **1** (5.0 g, 0.02 mol) was added to a solution of KOH (1.3 g, 0.024 mol) in anhydrous EtOH (160 mL). A solution of CS_2_ (2.0 g, 0.03 mol) in anhydrous EtOH (40 mL) was then added dropwise to the vigorously stirred mixture, which was refluxed for 6 hours. The solvent was removed under reduced pressure and the residue was dissolved in water (100 mL). After acidification to pH 5~6 with glacial acetic acid the crude product was isolated by filtration and recrystallized from ethanol/petroleum ether to afford 5.5 g of pure **5** as white crystals; m.p. 203-204 °C; yield: 94%; ^1^H-NMR δ (CDCl_3_): 2.50 (s, 1H, SH), 2.55 (s, 3H, 5-CH_3_), 2.65 (s, 3H, 7-CH_3_), 4.65 (s, 2H, SCH_2_), 7.15 (s, 1H, 6-H); Anal. calcd. for C_10_H_10_N_6_OS_2_ (294.34): C, 40.82; H, 3.40; N, 28.57; Found: C, 40.67; H, 3.51; N, 28.68.

### 2-(5,7-Dimethyl-1,2,4-triazolo[1,5-a]pyrimidine-2-thiomethyl)-5-alkylthio-1,3,4-oxadiazoles ***6a~b***

To a stirred solution of **5** (1.5 g, 5.1 mmol) and sodium hydroxide (0.2 g, 5.6 mmol) in water (15 mL), a mixture of a substituted benzyl chloride (5.6 mmol) and methanol (5 mL) was added dropwise. The resulting mixture was stirred at room temperature for 2 hours. The precipitate formed was filtered off and recrystallized from petroleum ether/acetone to give **6** in good yields.

**6a:** (R = *p*-NO_2_C_6_H_4_CH_2_): m.p. 132~133 ^o^C; yield 56.3%; ^1^H-NMR δ (CDCl_3_): 2.58 (s, 3H, 5-CH_3_), 2.65 (s, 3H, 7-CH_3_), 4.41 (s, 2H, CH_2_C_6_H_4_), 4.64 (s, 2H, SCH_2_), 6.72 (s, 1H, 6-H), 7.51-8.08 (q, 4H, Ar-H); MS (*m/z*): 429 (M^+^, 1), 293 (4), 262 (15), 261 (100), 221 (3), 219 (7), 193 (11), 180 (6), 149 (5), 148 (2), 136 (4), 108 (23), 107 (14); Anal. calcd. for C_17_H_15_N_7_O_3_S_2_ (429.47): C, 47.55; H, 3.50; N, 22.84; Found: C, 48.34; H, 3.96; N, 22.97.

**6b:** (R = *o*-ClC_6_H_4_CH_2_): m.p. 133~135 ^o^C; yield 75%; ^1^H-NMR δ (CDCl_3_): 2.65 (s, 3H, 5-CH_3_), 2.72 (s, 3H, 7-CH_3_), 4.38 (s, 2H, CH_2_C_6_H_4_), 4.72 (s, 2H, SCH_2_), 6.77 (s, 1H, 6-H), 7.23-7.39 (q, 4H, Ar-H); Anal. calcd. for C_17_H_15_N_6_OClS_2_ (418.91): C, 48.69; H, 3.58; N, 20.05; Found: C, 48.81; H, 3.75; N, 20.43.

### 2-(5,7-Dimethyl-1,2,4-triazolo[1,5-a]pyrimidine-2-thiomethyl)-5-phenyl-1,3,4-oxadiazole *(**7**)*

A mixture of **1** (1.0g, 4.0 mmol), benzoic acid (0.55 mL, 6 mmol) and POCl_3_ (5 mL) was refluxed for 6 hours. After cooling to room temperature, the mixture was poured into crushed ice and filtered. The solid was washed with sodium hydroxide solution (5%) and water (x 3) and recrystallized from EtOH to afford yellow crystals (0.9 g, 66% yield); m.p. 171-173 ^o^C; ^1^H-NMR δ (CDCl_3_): 2.64 (s, 3H, 5-CH_3_), 2.73 (s, 3H, 7-CH_3_), 4.82 (s, 2H, SCH_2_), 6.76 (s, 1H, 6-H), 7.47-8.01 (m, 5H, Ar-H); Anal. calcd. for C_16_H_14_N_6_OS: C, 56.80; H, 4.14; N, 24.85; Found: C, 57.13; H, 3.87; N, 24.69.

### 2-(5,7-dimethyl-1,2,4-triazolo[1,5-a]pyrimidine-2-thiomethyl)-1,3,4-thiadiazol-5-thiol *(**8**)*

To a solution of potassium hydroxide (4.68 g, 0.08 mol) and 2-thioacetohydrazide-5,7-dimethyl- 1,2,4-triazolo[1,5-a]pyrimidine (**1**, 15.1 g, 0.09 mol) in ethanol (250 mL), carbon disulfide (10 mL, 0.16 mol) was added dropwise at room temperature over a period of half an hour. The resulting mixture was stirred for 12 hours at room temperature and the precipitate formed was collected by filtration. After washing with ethanol and ethyl ether, the salt formed was added to concentrated H_2_SO_4_ (90 mL). The mixture was stirred at room temperature for 4 hours, poured into crushed ice (250 g) and the precipitate formed was filtered off and dissolved in NaOH solution (250 mL, 2.4 g). After filtration the filtrate was acidified with HCl, and the precipitate thus formed was filtered off and crystallized from ethanol to give pure **8** as yellow crystals (42%); m.p. 217~219 ^o^C; ^1^H-NMR δ (CD_3_COCD_3_): 2.60 (s, 3H, CH_3_), 2.76 (s, 3H, CH_3_), 4.67 (s, 2H, SCH_2_), 7.09 (s, 1H, CH), 13.89 (s, 1H, SH); Anal. calcd. for C_10_H_10_N_6_S_3_ (310.40): C, 38.66; H, 3.22; N, 27.06; Found: C, 38.43; H, 3.51; N, 27.40.

### 2-(5,7-dimethyl-1,2,4-triazolo[1,5-a]pyrimidine-2-thiomethyl)-5-alkylthio-1,3,4-thiadiazoles ***9a~c***

To a stirred solution of **8** (1.6 g, 5.1 mmol) and sodium hydroxide (0.2 g, 5.6 mmol) in water (15 mL), a mixture of substituted benzyl chloride (5.1 mmol) in methanol (5 mL) was added dropwise. The resulting mixture was stirred at room temperature for 2.5 hours. The precipitate formed was filtered off and recrystallized from petroleum ether/acetone to give **9** in fair to good yields.

**9**a (R = C_6_H_5_CH_2_): m.p. 118~120 ^o^C; yield 86%; ^1^H-NMR δ (CDCl_3_): 2.65 (s, 3H, CH_3_), 2.73 (s, 3H, CH_3_), 4.50 (s, 2H, SCH_2_), 4.84 (s, 2H, SCH_2_), 6.78 (s, 1H, CH), 7.25~7.38 (m, 5H, C_6_H_5_); MS (*m/z*): 400 (M^+^, 9), 309 (1), 237 (8), 181 (11), 180 (7), 149 (9), 148 (9), 91 (100), 67 (17); Anal. calcd. for C_17_H_16_N_6_S_3_ (400.53): C, 50.93; H, 3.99; N, 17.98; Found: C, 51.17; H, 4.26; N, 27.65.

**9**b (R = *p*-NO_2_C_6_H_4_CH_2_): m.p. 145~146 ^o^C; yield 68%; ^1^H-NMR δ (CDCl_3_): 2.65 (s, 3H, CH_3_), 2.72 (s, 3H, CH_3_), 4.56 (s, 2H, SCH_2_), 4.83 (s, 2H, SCH_2_), 6.78 (s, 1H, CH), 7.56~8.14 (m, 4H, C_6_H_4_); MS (*m/z*): 446 (M^+^, 7), 309 (10), 237 (25), 219 (12), 149 (26), 148 (20), 136 (15), 107 (60), 46 (24), 32 (100); Anal. calcd. for C_17_H_15_N_7_O_2_S_3_ (445.53): C, 45.79; H, 3.37; N, 21.99. Found: C, 45.97; H, 3.65; N, 22.33.

**9**c (R = *p*-ClC_6_H_4_CH_2_): m.p. 146~147^o^C; yield 44%; ^1^H-NMR δ (CDCl_3_): 2.65 (s, 3H, CH_3_), 2.72 (s, 3H, CH_3_), 4.45 (s, 2H, SCH_2_), 4.84 (s, 2H, SCH_2_), 6.78 (s, 1H, CH), 7.24~7.34 (m, 4H, C_6_H_4_); MS (*m/z*): 435 (M^+^, 7), 309 (4), 237 (10), 193 (3), 181 (19), 125 (100), 108 (14), 107 (25); Anal. calcd. for C_17_H_15_N_6_ClS_3_ (434.97): C, 46.89; H, 3.45; N, 19.31; Found: C, 46.61; H, 3.23; N, 19.64.
